# Supplementary material for: Terrestrial wildlife as indicators of microplastic pollution in western Thailand
Source: PeerJ. 2024 May 20;12:e17384. doi: 10.7717/peerj.17384 (PMC11114113; doi:10.7717/peerj.17384)
Supplement: Supplemental Information 1 [file peerj-12-17384-s001.docx]

**Supplementary Table 1:**

**The list of species, numbers of specimen, quantities of microplastics classified by species and groups of terrestrial vertebrates, comparing between inside and outside protected areas, and the whole study areas in western Thailand.**

| **Groups/Species** | **No. Sample** | | | **MPs (ind^-1^) (mean±SD)** | | | **MPs (g^-1^) (mean±SD)** | | |
| --- | --- | --- | --- | --- | --- | --- | --- | --- | --- |
|  | **Total** | **Inside PAs** | **Outside PAs** | **Total** | **Inside PAs** | **Outside PAs** | **Total** | **Inside PAs** | **Outside PAs** |
| **Amphibians** | **8** | **2** | **6** | **4.25±7.50** | **3.00±4.24** | **4.67±8.62** | **0.04±0.09** | **0.013±0.018** | **0.05±0.10** |
| 1. *Hoplobatrachus rugulosus* | 1 | 0 | 1 | 1 | - | 1 | 0.03 | - | 0.03 |
| 2. *Fejervarya limnocharis* | 1 | 0 | 1 | 22 | - | 22 | 0.25 | - | 0.25 |
| 3. *Duttaphrynus melanostictus* | 4 | 1 | 3 | 1.25±1.89 | 0 | 1.67±2.08 | 0.009±0.01 | 0 | 0.01±0.01 |
| 4. *Kaloula pulchra* | 2 | 1 | 1 | 3.00±4.00 | 6 | 0 | 0.01±0.02 | 0.03 | 0 |
| **Lizards and other reptiles** | **7** | **6** | **1** | **1.29±1.25** | **1.33±1.37** | **1** | **0.02±0.03** | **0.02±0.03** | **0.001** |
| 5. *Acanthosaura lepidogaster* | 1 | 1 | 0 | 0 | 0 | - | 0 | 0 | - |
| 6. *Calotes versicolor* | 1 | 1 | 0 | 1 | 1 | - | 0.08 | 0.08 | - |
| 7. Scincidae | 1 | 1 | 0 | 0 | 0 | - | 0 | 0 | - |
| 8. *Amyda cartilaginea* | 1 | 1 | 0 | 1 | 1 | - | 0.01 | 0.01 | - |
| 9. *Varanus salvator* | 1 | 0 | 1 | 1 | - | 1 | 0.001 | - | 0.001 |
| 10. *Gekko gecko* | 2 | 2 | 0 | 3.00±0.00 | 3.00±0.00 | - | 0.02±0.00 | 0.02±0.00 | - |
| **Snakes** | **60** | **29** | **31** | **3.52±3.20** | **3.38±2.96** | **3.65±3.46** | **0.05±0.09** | **0.03±0.04** | **0.07±0.12** |
| 11. *Chrysopelea ornata* | 2 | 1 | 1 | 1±0.00 | 1 | 1 | 0.01±0.009 | 0.02 | 0.01 |
| 12. *Trimeresurus barati* | 1 | 1 | 0 | 5 | 5 | - | 0.04 | 0.04 | - |
| 13. *Boiga siamensis* | 2 | 2 | 0 | 1.00±1.41 | 1.00±1.41 | - | 0.002±0.003 | 0.002±0.003 | - |
| 14. *Xenopeltis unicolor* | 4 | 1 | 3 | 1.50±0.58 | 2 | 1.33±0.58 | 0.01±0.002 | 0.01 | 0.01±0.002 |
| 15. *Pareas carinatus* | 13 | 5 | 8 | 3.85±3.26 | 1.60±0.89 | 5.25±3.45 | 0.14±0.17 | 0.11±0.002 | 0.21±0.19 |
| 16. *Pareas margaritophorus* | 4 | 2 | 2 | 3.25±1.89 | 4.00±2.83 | 2.50±0.71 | 0.09±0.01 | 0.10±0.002 | 0.08±0.01 |
| 17. *Oligodon taeniatus* | 1 | 1 | 0 | 1 | 1 | - | 0.13 | 0.13 | - |
| 18. *Ophiophagus hannah* | 1 | 1 | 0 | 1 | 1 | - | 0.03 | 0.03 | - |
| 19. *Coelognathus radiates* | 6 | 3 | 3 | 5.17±4.62 | 7.00±4.36 | 3.33±4.93 | 0.02±0.03 | 0.04±0.04 | 0.002±0.002 |
| 20. *Dryocalamus davidsonii* | 3 | 1 | 2 | 1.33±0.58 | 2 | 1.00±0.00 | 0.06±0.01 | 0.05 | 0.07±0.003 |
| 21. *Hypsiscopus plumbea* | 1 | 0 | 1 | 2 | - | 2 | 0.04 | - | 0.04 |
| 22. *Oligodon* spp. | 4 | 3 | 1 | 3.25±4.03 | 4.00±4.58 | 1 | 0.014±0.01 | 0.02±0.01 | 0.02 |
| 23. *Dendrelaphis* spp. | 3 | 1 | 2 | 6.67±4.51 | 2 | 9.00±2.83 | 0.03±0.01 | 0.02 | 0.03±0.01 |
| 24. *Rhabdophis subminiatus* | 2 | 0 | 2 | 7.50±6.36 | - | 7.50±6.36 | 0.02±0.003 | - | 0.02±0.003 |
| 25. *Lycodon capucinus* | 2 | 1 | 1 | 2.50±0.71 | 3 | 2 | 0.06±0.03 | 0.04 | 0.08 |
| 26. *Ptyas* spp. | 7 | 4 | 3 | 4.14±3.29 | 6.25±2.75 | 1.33±0.58 | 0.02±0.03 | 0.03±0.04 | 0.01±0.002 |
| 27. *Ptyas mucosa* | 1 | 0 | 1 | 4 | 0 | 4 | 0.01 | - | 0.01 |
| 28. *Plagiopholis nuchalis* | 1 | 1 | 0 | 2 | 2 | - | 0.04 | 0.04 | - |
| 29. *Trimeresurus kanburiensis* | 1 | 1 | 0 | 3 | 3 | - | 0.004 | 0.004 | - |
| 30. *Daboia siamensis* | 1 | 0 | 1 | 3 | - | 3 | 0.03 | - | 0.03 |
| **Birds** | **15** | **5** | **10** | **2.73±3.22** | **2.20±1.92** | **3.00±3.77** | **0.03±0.05** | **0.07±0.06** | **0.02±0.02** |
| 31. Unknown#1 | 1 | 1 | 0 | 3 | 3 | - | 0.05 | 0.05 | - |
| 32. Unknown#2 | 1 | 1 | 0 | 0 | 0 | - | 0 | 0 | - |
| 33. *Glaucidium cuculoides* | 1 | 0 | 1 | 1 | - | 1 | 0.01 | - | 0.01 |
| 34. *Acridotheres grandis* | 2 | 0 | 2 | 2.00±1.41 | - | 2.00±1.41 | 0.01±0.002 | - | 0.01±0.002 |
| 35. *Phylloscopus inornatus* | 1 | 1 | 0 | 2 | 2 | - | 0.17 | 0.17 | - |
| 36. *Centropus sinensis* | 3 | 0 | 3 | 2.00±1.73 | - | 2.00±1.73 | 0.004±0.001 | - | 0.004±0.001 |
| 37. *Copsychus saularis* | 1 | 0 | 1 | 13 | - | 13 | 0.03 | - | 0.029 |
| 38. *Luscinia calliope* | 1 | 1 | 0 | 1 | 1 |  | 0.05 | 0.05 | - |
| 39. *Coracias benghalensis* | 1 | 0 | 1 | 0 | - | 0 | 0 | - | 0 |
| 40. *Passer montanus* | 1 | 1 | 0 | 5 | 5 | - | 0.06 | 0.06 | - |
| 41. *Pycnonotus aurigaster* | 2 | 0 | 2 | 3.00±1.41 | - | 3.00±1.41 | 0.04±0.04 | - | 0.04±0.04 |
| **Mammals** | **2** | **1** | **1** | **2.00±0.00** | **2** | **2** | **0.01±0.004** | **0.009** | **0.003** |
| 42. *Callosciurus caniceps* | 1 | 0 | 1 | 2 | - | 2 | 0.003 | - | 0.003 |
| 43. Muridae | 1 | 1 | 0 | 2 | 2 | - | 0.01 | 0.01 | - |
| **Only all terrestrial carcasses** | **92** | **43** | **49** | **3.25±3.63** | **2.91±2.73** | **3.55±4.28** | **0.05±0.08** | **0.03±0.04** | **0.06±0.11** |
| **Tadpoles** | **44** | **26** | **18** | **2.00±3.13** | **0.92±1.13** | **3.56±4.31** | **12.88±30.79** | **1.98±6.06** | **28.63±43.59** |
| 1. *Clinotarus penelops* | 20 | 12 | 8 | 1.00±0.97 | 0.91±1.16 | 1.13±0.64 | 0.82±1.15 | 0.51±0.95 | 1.30±1.32 |
| 2. *Microhyla berdmorei* | 5 | - | 5 | 2.60±1.52 | - | 2.60±1.52 | 26.00±15.17 | - | 26.00±15.17 |
| 3. *Limnonectes* sp. | 3 | 3 | - | 1.33±1.53 | 1.33±1.53 | - | 13.33±15.27 | 13.33±15.27 | - |
| 4. *Leptobrachium* sp. | 3 | 3 | - | 1.33±1.53 | 1.33±1.53 | - | 0.36±0.41 | 0.36±0.41 | - |
| 6. *Rhacophorus bipunctatus* | 5 | 5 | - | 1.00±1.00 | 1.00±1.00 | - | 0.87±0.90 | 0.87±0.90 | - |
| 7. *Fejervarya limnocharis* | 8 | 3 | 5 | 5.25±6.18 | 0.00±0.00 | 8.40±5.81 | 46.88±59.94 | 0.00±0.00 | 75.00±60.40 |
| **Total** | **136** | **69** | **67** | **2.85±3.52** | **2.16±2.45** | **3.55±4.25** | **4.20±18.39** | **0.77±3.80** | **7.73±25.54** |
