# Supplementary material for: Terrestrial wildlife as indicators of microplastic pollution in western Thailand
Source: PeerJ. 2024 May 20;12:e17384. doi: 10.7717/peerj.17384 (PMC11114113; doi:10.7717/peerj.17384)
Supplement: Supplemental Information 2 [file peerj-12-17384-s002.docx]

**Supplementary Table 2:**

**Quantities of microplastics classified by types, colors, and sizes in vertebrate carcasses and tadpole, comparing between inside and outside protected areas, and the whole study areas in western Thailand**

| **Types/Colors/Sizes of MPs** | **No. of MPs** | | | **MPs (ind^-1^) (mean±SD)** | | | **MPs (g^-1^) (mean±SD)** | | |
| --- | --- | --- | --- | --- | --- | --- | --- | --- | --- |
|  | **Inside PAs** | **Outside PAs** | **Total** | **Inside PAs** | **Outside PAs** | **Total** | **Inside PAs** | **Outside PAs** | **Total** |
| **Total by Types** |  |  |  |  |  |  |  |  |  |
| Fibers | 118 | 180 | 298 | 1.71±2.09 | 2.69±2.95 | 2.19±2.59 | 0.05±0.07 | 0.08±0.09 | 0.07±0.08 |
| Films | - | 1 | 1 | - | 0.02±0.12 | 0.01±0.09 | - | 0.001±0.04 | 0.0002±0.003 |
| Foams | - | 2 | 2 | - | 0.03±017 | 0.02±0.12 | - | 0.001±0.005 | 0.001±0.004 |
| Fragments | 31 | 55 | 86 | 0.44±1.15 | 0.82±2.11 | 0.63±1.69 | 0.01±0.04 | 0.03±0.07 | 0.02±0.05 |
| **Carcasses** |  |  |  |  |  |  |  |  |  |
| Fibers | 95 | 135 | 230 | 0.07±0.08 | 0.09±0.10 | 2.50±2.67 | 0.07±0.08 | 0.09±0.10 | 0.08±0.09 |
| Films | - | 1 | 1 | - | 0.001±0.005 | 0.01±0.10 | - | 0.0006±0.005 | 0.003±0.003 |
| Foams | - | 1 | 1 | - | 0.0006±0.005 | 0.01±0.11 | - | 0.001±0.005 | 0.0003±0.003 |
| Fragments | 30 | 37 | 67 | 0.02±0.04 | 0.02±0.05 | 0.73±1.56 | 0.02±0.04 | 0.02±0.05 | 0.03±0.05 |
| **Tadpoles** |  |  |  |  |  |  |  |  |  |
| Fibers | 23 | 45 | 68 | 0.03±0.04 | 0.08±0.09 | 1.56±2.07 | 0.03±0.04 | 0.08±0.09 | 0.05±0.07 |
| Films | - | - | - | - | - | - | - | - | - |
| Foams | - | 1 | 1 | - | 0.002±0.007 | 0.02±0.15 | - | 0.002±0.007 | 0.001±0.005 |
| Fragments | 1 | 18 | 19 | 0.001±0.006 | 0.03±0.09 | 0.43±1.95 | 0.001±0.006 | 0.03±0.09 | 0.01±0.06 |
| **Total by Colors** | **149** | **238** | **387** |  |  |  |  |  |  |
| Black | 36 | 40 | 76 | 0.52±1.11 | 0.60±1.13 | 0.56±1.11 | 1±2×10^-3^ | 1.00±2.00×10^-3^ | 1.00±2.00×10^-3^ |
| Blue | 60 | 102 | 162 | 0.87±1.67 | 1.52±2.31 | 1.19±2.03 | 2±4×10^-3^ | 3.00±5.00×10^-3^ | 3.00±4.00×10^-3^ |
| Brown | 3 | 10 | 13 | 0.04±0.21 | 0.15±0.78 | 0.10±0.57 | 0.9±4×10^-4^ | 0.30±2.00×10^-3^ | 0.20±1.00×10^-3^ |
| Green | 6 | 3 | 9 | 0.09±0.33 | 0.05±0.21 | 0.07±0.28 | 1.86±7.09×10^-4^ | 0.96±4.46×10^-4^ | 1.00±6.00×10^-4^ |
| Gray | 8 | 9 | 17 | 0.12±0.40 | 0.13±0.39 | 0.13±0.39 | 2.48±8.63×10^-4^ | 2.87±8.24×10^-4^ | 3.00±8.00×10^-4^ |
| Light blue | 3 | 11 | 14 | 0.04±0.21 | 0.16±0.45 | 0.10±0.35 | 0.93±4.39×10^-4^ | 3.51±9.56×10^-4^ | 2.00±8.00×10^-4^ |
| Orange | 3 | 3 | 6 | 0.04±0.21 | 0.05±0.21 | 0.04±0.21 | 0.93±4.39×10^-4^ | 0.96±4.46×10^-4^ | 0.9±4.00×10^-4^ |
| Purple | 6 | 16 | 22 | 0.09±0.37 | 0.24±0.55 | 0.16±0.48 | 1.86±7.99×10^-4^ | 0.51±1.18×10^-3^ | 0.4±1.00×10^-3^ |
| Red | 9 | 7 | 16 | 0.13±0.48 | 0.11±0.35 | 0.12±0.42 | 0.28±1.03×10^-3^ | 2.24±7.57×10^-4^ | 3±9.00×10^-4^ |
| Transparent | 5 | 11 | 16 | 0.07±0.26 | 0.16±0.51 | 0.12±0.41 | 1.55±5.59×10^-4^ | 0.35±1.09×10^-4^ | 3±9.00×10^-4^ |
| White | 8 | 22 | 30 | 0.12±0.40 | 0.33±1.09 | 0.22±0.82 | 2.48±8.63×10^-4^ | 0.7±2.34×10^-3^ | 0.5±2.00×10^-3^ |
| Yellow | 2 | 4 | 6 | 0.03±0.24 | 0.06±0.24 | 0.04±0.24 | 0.62±5.15×10^-4^ | 1.28±5.11×10^-4^ | 0.9±5.00×10^-4^ |
| **Animal carcasses** | **125** | **174** | **299** |  |  |  |  |  |  |
| Black | 24 | 26 | 50 | 0.56±1.20 | 0.53±1.24 | 0.54±1.22 | 1.19±2.57×10^-3^ | 1.13±2.66×10^-3^ | 1.2±2.6×10^-3^ |
| Blue | 52 | 84 | 136 | 1.21±1.99 | 1.71±2.46 | 1.48±2.26 | 2.59±5.27×10^-3^ | 3.67±5.26×10^-3^ | 3.2±4.8×10^-3^ |
| Brown | 3 | 7 | 10 | 0.07±0.26 | 0.14±0.87 | 0.11±0.65 | 1.5±5.5×10^-4^ | 0.3±1.9×10^-3^ | 2.3±1.4×10^-4^ |
| Green | 6 | 1 | 7 | 0.14±0.41 | 0.02±0.14 | 0.08±0.31 | 3.0±8.8×10^-4^ | 0.4±3.1×10^-4^ | 1.6±6.5×10^-4^ |
| Gray | 6 | 4 | 10 | 0.14±0.47 | 0.08±0.34 | 0.11± 0.41 | 3.0±10.0×10^-4^ | 1.8±7.4×10^-4^ | 2.3±8.7×10^-4^ |
| Light blue | 3 | 9 | 12 | 0.07±0.26 | 0.18±0.49 | 0.10±0.35 | 1.5±5.5×10^-4^ | 0.4±1.0×10^-3^ | 2.8±8.5×10^-4^ |
| Orange | 3 | 2 | 5 | 0.07±0.26 | 0.04±0.20 | 0.05±0.23 | 1.5±5.5×10^-4^ | 0.9±4.3×10^-4^ | 1.2±4.9×10^-4^ |
| Purple | 6 | 13 | 19 | 0.14±0.47 | 0.27±0.57 | 0.21±0.53 | 3.0±10.0×10^-4^ | 0.6±1.2×10^-3^ | 0.4±1.1×10^-3^ |
| Red | 9 | 6 | 15 | 0.21±0.60 | 0.12±0.39 | 0.16±0.50 | 0.5±1.3×10^-3^ | 2.6±8.3×10^-4^ | 0.4±1.1×10^-3^ |
| Transparent | 4 | 5 | 9 | 0.09±0.29 | 0.10±0.31 | 0.10±0.30 | 2.0±6.3×10^-4^ | 2.2±6.5×10^-4^ | 2.1±6.4×10^-4^ |
| White | 7 | 14 | 21 | 0.16±0.49 | 0.29±0.82 | 0.23±0.68 | 3.48±1.00×10^-4^ | 0.6±1.8×10^-3^ | 0.5×1.510^-3^ |
| Yellow | 2 | 3 | 5 | 0.05±0.31 | 0.06±0.24 | 0.05±0.27 | 1.00±6.52×10^-4^ | 1.3±5.2×10^-4^ | 1.2±5.8×10^-4^ |
| **Tadpoles** | **24** | **64** | **88** |  |  |  |  |  |  |
| Black | 12 | 14 | 26 | 0.46±0.95 | 0.78± 0.73 | 0.59±0.87 | 0.99±2.03×10^-4^ | 1.7±1.6×10^-3^ | 1.3±1.9×10^-3^ |
| Blue | 8 | 18 | 26 | 0.31±0.62 | 1.00±1.82 | 0.59±1.28 | 0.66±1.32×10^-4^ | 2.1±3.9×10^-3^ | 1.3±2.7×10^-3^ |
| Brown | 0 | 3 | 3 | - | 0.17±0.52 | 0.07±0.33 | - | 0.4±1.1×10^-3^ | 1.5±7.1×10^-4^ |
| Green | 0 | 2 | 2 | - | 0.11±0.32 | 0.05± 0.21 | - | 2.4±6.9×10^-4^ | 1.0±4.5×10^-4^ |
| Gray | 2 | 5 | 7 | 0.08±0.27 | 0.28±0.46 | 0.16±0.37 | 1.65±5.81×10^-4^ | 5.9±9.9×10^-4^ | 3.4±7.9×10^-4^ |
| Light blue | 0 | 2 | 2 | - | 0.11±0.32 | 0.10±0.35 | - | 2.38±6.91×10^-4^ | 1.0×4.510^-4^ |
| Orange | 0 | 1 | 1 | - | 0.06±0.24 | 0.02±0.15 | - | 1.19±5.04×10^-4^ | 0.5±9.2×10^-4^ |
| Purple | 0 | 3 | 3 | - | 0.17±0.51 | 0.07±0.33 | - | 0.36±1.10×10^-3^ | 1.5±7.1×10^-4^ |
| Red | 0 | 1 | 1 | - | 0.06±0.24 | 0.02±0.15 | - | 1.19±5.04×10^-4^ | 0.5±3.2×10^-4^ |
| Transparent | 1 | 6 | 7 | 0.04±0.20 | 0.33±0.84 | 0.16±0.57 | 0.83±4.19×10^-4^ | 0.71±1.80×10^-3^ | 0.3±1.2×10^-3^ |
| White | 1 | 8 | 9 | 0.16±0.49 | 0.04±0.20 | 0.21±1.07 | 0.83±4.19×10^-4^ | 9.51±3.54×10^-3^ | 0.4±2.29×10^-3^ |
| Yellow | 0 | 1 | 1 | - | 0.06±0.24 | 0.02±0.15 | - | 1.18±5.04×10^-4^ | 0.49±3.2×10^-4^ |
| **Total by Sizes** | **149** | **238** | **387** |  |  |  |  |  |  |
| Very small (<50 µm) | 0 | 1 | 1 | - | 0.02±0.12 | 0.01±0.09 | - | 0.46±3.77×10^-4^ | 0.23±2.64×10^-4^ |
| Small (50-500 µm) | 43 | 74 | 117 | 0.62±1.18 | 1.10±2.49 | 0.86±1.94 | 1.92±3.63×10^-3^ | 3.40±7.67×10^-3^ | 2.65±5.99×10^-3^ |
| Slightly small (500-1000 µm) | 32 | 54 | 86 | 0.46±0.92 | 0.81±1.13 | 0.63±1.04 | 1.43±2.83×10^-3^ | 2.48±3.49×10^-3^ | 1.95±3.20×10^-3^ |
| Moderate (1000-2000 µm) | 45 | 63 | 108 | 0.65±1.05 | 0.94±1.24 | 0.79±1.16 | 2.01±3.25×10^-3^ | 3.00±3.83×10^-3^ | 2.45±3.56×10^-3^ |
| Slightly large (2000-3000 µm) | 11 | 20 | 31 | 0.16±0.37 | 0.30±0.60 | 0.23±0.50 | 0.49±1.11×10^-3^ | 9.20±2.27×10^-4^ | 0.70±1.55×10^-3^ |
| Large (3000-4000 µm) | 10 | 15 | 25 | 0.15±0.43 | 0.22±0.49 | 0.18±0.46 | 0.15±0.43 | 0.22±0.49 | 0.57±1.41×10^-3^ |
| Very large (4000-5000 µm) | 4 | 4 | 8 | 0.06±0.24 | 0.06±0.24 | 0.06±0.24 | 0.06±0.24 | 0.06±0.24 | 1.81±7.28×10^-4^ |
| Plastic debris (>5000 µm) | 4 | 7 | 11 | 0.06±0.24 | 0.10±0.35 | 0.08±0.30 | 0.06±0.24 | 0.10±0.35 | 2.49±9.23×10^-4^ |
| **Animal carcasses** | **125** | **174** | **299** |  |  |  |  |  |  |
| Very small (<50 µm) | - | 1 | 1 | - | 0.02±0.14 | 0.01±0.10 | - | 0.63±4.40×10^-4^ | 0.35±3.21×10^-4^ |
| Small (50-500 µm) | 39 | 54 | 93 | 0.91±1.38 | 1.10±2.21 | 1.01±1.86 | 2.80±4.24×10^-3^ | 3.40±6.81×10^-3^ | 3.12±5.73×10^-3^ |
| Slightly small (500-1000 µm) | 28 | 37 | 65 | 0.65±1.04 | 0.76±1.09 | 0.71±1.06 | 2.01±3.22×10^-3^ | 2.33±3.36×10^-3^ | 2.18±3.28×10^-3^ |
| Moderate (1000-2000 µm) | 36 | 46 | 82 | 0.84±1.25 | 0.94±1.25 | 0.89±1.24 | 2.58±3.86×10^-3^ | 2.90±3.85×10^-3^ | 2.75±3.83×10^-3^ |
| Slightly large (2000-3000 µm) | 7 | 13 | 20 | 0.16±0.37 | 0.27±0.61 | 0.22±0.51 | 0.50±1.15×10^-3^ | 0.82±1.86×10^-3^ | 0.67±1.59×10^-3^ |
| Large (3000-4000 µm) | 8 | 13 | 21 | 0.19±0.50 | 0.27±0.53 | 0.23±0.52 | 5.73±1.54×10^-4^ | 0.82±1.64×10^-3^ | 0.70±1.59×10^-3^ |
| Very large (4000-5000 µm) | 3 | 4 | 7 | 0.70±0.26 | 0.08±0.28 | 0.08±0.27 | 2.15±7.94×10^-4^ | 2.52±8.53×10^-4^ | 2.34±8.22×10^-4^ |
| Plastic debris (>5000 µm) | 4 | 6 | 10 | 0.09±0.29 | 0.12±0.39 | 0.11±0.35 | 2.87±9.06×10^-4^ | 0.38±1.20×10^-3^ | 0.34±1.07×10^-3^ |
| **Tadpoles** | **24** | **64** | **88** |  |  |  |  |  |  |
| Very small (<50 µm) | 0 | 0 | 0 | - | - | - | - | - | - |
| Small (50-500 µm) | 4 | 20 | 24 | 0.15±0.46 | 1.11±3.20 | 0.55±2.10 | 0.47±1.43×10^-3^ | 3.42±9.85×10^-3^ | 1.68±6.46×10^-3^ |
| Slightly small (500-1000 µm) | 4 | 17 | 21 | 0.15±0.54 | 0.94±1.26 | 0.48±0.98 | 0.47±1.67×10^-3^ | 2.91±3.88×10^-3^ | 1.47±3.01×10^-3^ |
| Moderate (1000-2000 µm) | 9 | 17 | 26 | 0.35±0.49 | 0.94±1.26 | 0.59±0.92 | 1.07±1.50×10^-3^ | 2.91±3.88×10^-3^ | 1.82±2.85×10^-3^ |
| Slightly large (2000-3000 µm) | 4 | 7 | 11 | 0.15±0.37 | 0.39±0.61 | 0.25 0.49± | 0.47±1.13×10^-3^ | 1.20±1.87×10^-3^ | 0.77±1.51×10^-3^ |
| Large (3000-4000 µm) | 2 | 2 | 4 | 0.08±0.27 | 0.11±0.32 | 0.91±0.29 | 2.37±8.37×10^-4^ | 3.42±9.97×10^-4^ | 2.80±8.96×10^-4^ |
| Very large (4000-5000 µm) | 1 | 0 | 1 | 0.04±0.20 | - | 0.02±0.15 | 1.19±6.04×10^-4^ | - | 0.38±4.65×10^-4^ |
| Plastic debris (>5000 µm) | 0 | 1 | 1 | - | 0.06±0.24 | 0.02± 0.15 | - | 1.71±7.26×10^-4^ | 0.38±4.65×10^-4^ |
